# A New Role for *Helicobacter pylori* Urease: Contributions to Angiogenesis

**DOI:** 10.3389/fmicb.2017.01883

**Published:** 2017-09-27

**Authors:** Deiber Olivera-Severo, Augusto F. Uberti, Miguel S. Marques, Marta T. Pinto, Maria Gomez-Lazaro, Céu Figueiredo, Marina Leite, Célia R. Carlini

**Affiliations:** ^1^Center of Biotechnology, Universidade Federal Rio Grande do Sul, Porto Alegre, Brazil; ^2^Biology Department, Universidade Regional Integrada do Alto Uruguai e das Missões, São Luiz Gonzaga, Brazil; ^3^Institute of Biology, Universidade Federal de Pelotas, Pelotas, Brazil; ^4^i3S, Instituto de Investigação e Inovação em Saúde, University of Porto, Porto, Portugal; ^5^Ipatimup-Institute of Molecular Pathology and Immunology of the University of Porto, Porto, Portugal; ^6^Faculty of Medicine of the University of Porto, Porto, Portugal; ^7^INEB - Instituto Nacional de Engenharia Biomédica, University of Porto, Porto, Portugal; ^8^Brain Institute (BRAINS-InsCer), Pontifícia Universidade Católica do Rio Grande do Sul, Porto Alegre, Brazil

**Keywords:** *Helicobacter pylori*, urease, endocytosis, vasculogenesis, angiogenesis

## Abstract

*Helicobacter pylori* is a pathogen involved in gastric diseases such as ulcers and carcinomas. *H. pylori’s* urease is an important virulence factor produced in large amounts by this bacterium. In previous studies, we have shown that this protein is able to activate several cell types like neutrophils, monocytes, platelets, endothelial cells, and gastric epithelial cells. Angiogenesis is a physiological process implicated in growth, invasion and metastization of tumors. Here, we have analyzed the angiogenic potential of *H. pylori* urease (HPU) in gastric epithelial cells. No cytotoxicity was observed in AGS, Kato-III, and MKN28 gastric cell lines treated with 300 nM HPU, as evaluated by the 3-(4,5-Dimethylthiazol-2-yl)-2,5-diphenyltetrazolium bromide (MTT) assay. As we previously reported in neutrophils, treatment with 300 nM HPU also had an anti-apoptotic effect in gastric epithelial cells leading to a 2.2-fold increase in the levels of Bcl-X_L_ after 6 h, and a decrease of 80% in the content of BAD, after 48 h, two mitochondrial proteins involved in regulation of apoptosis. Within 10 min of exposure, HPU is rapidly internalized by gastric epithelial cells. Treatment of the gastric cells with methyl-β-cyclodextrin abolished HPU internalization suggesting a cholesterol-dependent process. HPU induces the expression of pro-angiogenic factors and the decrease of expression of anti-angiogenic factors by AGS cells. The angiogenic activity of HPU was analyzed using *in vitro* and *in vivo* models. HPU induced formation of tube-like structures by human umbilical vascular endothelial cells in a 9 h experiment. In the chicken embryo chorioallantoic membrane model, HPU induced intense neo-vascularization after 3 days. In conclusion, our results indicate that besides allowing bacterial colonization of the gastric mucosa, *H. pylori*’s urease triggers processes that initiate pro-angiogenic responses in different cellular models. Thus, this bacterial urease, a major virulence factor, may also play a role in gastric carcinoma development.

## Introduction

*Helicobacter pylori* is a Gram-negative bacterium that infects more than half of the world’s population and it is the major cause of gastroduodenal diseases, such as gastritis, peptic ulcers, and gastric cancer (IARC, 1994). *H. pylori* infection was responsible for 770,000 new cases of gastric cancer worldwide in 2012 (Plummer et al., 2016). Gastric cancer is the third most common cause of cancer-related deaths worldwide according to GLOBOCAN 2012 database, and risk factors include smoking, obesity, diet and bacterial infections, most importantly, *H. pylori* infection (Ferlay et al., 2013).

This bacterium produces large amounts of urease, an important virulence factor involved in a series of processes that allow bacteria to colonize and induce a strong inflammatory response in the gastric epithelium. HPU enzymatic activity causes hydrolysis of urea into ammonia, thereby neutralizing the acid environment of the stomach (Marshall et al., 1990), leading to altered properties of the gastric mucous layer (Perrais et al., 2014). HPU is a large protein, consisting of a dodecameric organization of two subunits (UreA, 26.5 kDa; UreB, 61.7 kDa; Ha et al., 2001). Its smaller subunit UreA was found to localize in the nuclei of cultured gastric epithelial cells, leading to altered morphology of AGS cells (Lee et al., 2012, 2015). Additionally, both HPU subunits, UreA and UreB, were observed inside outer membrane vesicles secreted by *H. pylori* and vesicles derived from pathogenic bacteria are often involved in toxin delivery to the host (Olofsson et al., 2010). By a mechanism not yet fully understood, HPU is also involved in the dysregulation of gastric epithelial tight junctions (Wroblewski et al., 2009) and independently of its enzyme activity, it promotes activation of neutrophils both *in vivo* and *in vitro* conditions (Uberti et al., 2013; Carlini and Ligabue-Braun, 2016).

Angiogenesis, the formation of new blood vessels from the pre-existing vasculature, is essential for tumor growth, invasion, and metastatic dissemination and it plays a central role in the progression of gastric cancer by providing nutrients and oxygen (Hanahan and Weinberg, 2011; De Palma et al., 2017; Macedo et al., 2017). *H. pylori* infection can induce and modulate the synthesis of angiogenic and invasive factors in gastric cancer cells (Kitadai, 2010), and gastritis *H. pylori*-positive patients have an increased number of blood vessels in the gastric mucosa compared to *H. pylori*-negative patients (Yeo et al., 2006). Recently, Liu et al. (2016) showed that *H. pylori* colonization correlated to the depth of tumor invasion and higher stage metastasis. VEGF is overexpressed in *H. pylori*-positive patients and gastric cancer tumors (Okines et al., 2011). However, VEGF overexpression is a poor prognosis indicator of increased angiogenesis in gastric cancer, suggesting that different factors may be involved in this process (Pinto et al., 2017).

Here, we seek to investigate whether HPU plays a role in the angiogenesis process induced by *H. pylori*.

## Materials and Methods

### Cell Culture

Human AGS (ATCC^®^CRL-1739^TM^), AGS-Ecad (Oliveira et al., 2009), Kato-III (ATCC^®^HTB-103^TM^), MKN28 (JCRB0253^TM^) were maintained in RPMI 1640 medium with GlutaMAX^TM^ (Invitrogen, Thermo Fisher Scientific, Inc., Waltham, MA, United States) supplemented with 10% fetal bovine serum (FBS, HyClone^TM^, GE Healthcare Life Sciences, Logan, UT, United States), 200 IU/mL penicillin G-200 μg/mL streptomycin sulfate (Invitrogen) at 37°C under 5% CO_2_ humidified atmosphere. Human umbilical vein endothelial cells (HUVECs; HUV-EC-C, ATCC^®^CRL1730^TM^) were maintained in Medium 199 (M199) with Earle’s salts, stable glutamine, and 25 mM HEPES buffer (Biowest, Nuaillé, France) supplemented with 10% FBS, 100 IU-100 μg/mL Penicillin G-Streptomycin Sulfate (Gibco), 100 μg/mL Heparin (Sigma–Aldrich, St. Louis, MO, United States), and 30 μg/mL Endothelial Mitogen (ECGS) (BioMedical Technologies, Inc., Stoughton, MA, United States), in gelatin-coated (Sigma–Aldrich, St. Louis, MO, United States) tissue-culture Petri dishes (TPP^®^ Plastic Products AG, Trasadingen, Switzerland), at 37°C under 5% CO_2_ humidified atmosphere.

### Bacterial Strain and Growth Conditions

Bacteria were grown for 48 h at 37°C under a microaerophilic atmosphere (GENbox microaer; bioMérieux S.A., Marcy l’Etoile, France) in Trypticase^TM^ Soy Agar with 5% sheep blood (TSAII; Becton, Dickinson and Company, Franklin Lakes, NJ, United States). Experiments were performed with *H. pylori* strain 26695 (ATCC^®^700392^TM^; *cag* PAI^+^).

### HPU Purification

*Helicobacter pylori* bacteria grown for 48 h in TSAII (40 plates) were harvested in phosphate buffer (20 mM NaH_2_PO_4_, pH 7.5) and lysed by ultrasound (Ultrasonic Homogenizer 4710; 10 pulses of 30 s in ice bath). After centrifugation (20 min, 20,000 × *g*, 4°C), the supernatant was processed according to a previously published protocol to obtain purified HPU (Wassermann et al., 2010). Protein purity was assessed by gel electrophoresis in 10% polyacrylamide gels containing 0.1% sodium dodecyl sulfate (SDS-PAGE).

### Immunofluorescence and Microscopy

Immunofluorescence studies and analysis by laser scanning confocal microscopy were performed using a PL APO 63× NA 1.40 oil objective (Spectral Confocal Microscope Leica TCS-SP5; Leica Microsystems, Mannheim, Germany). The images were combined and merged using ImageJ (Schneider et al., 2012) with the plugin Bio-Formats (Linkert et al., 2010). For immunocytochemistry, after treatment with 100 nM HPU for 5, 15, and 45 min, the AGS cells were fixed with 4% paraformaldehyde (Polysciences, Warrington, PA, United States), washed with phosphate buffered saline (PBS), and permeabilized with ice-cold 100% methanol. Cells were incubated with the primary rabbit polyclonal antibody anti-UreB and mouse monoclonal antibody anti-Early Endosome Antigen 1 (EEA1) (Santa Cruz Biotechnology, Dallas, TX, United States). The secondary antibodies Alexa Fluor 488 goat anti-rabbit IgG and Alexa Fluor 596 goat anti-mouse (Thermo Fisher Scientific) were used to monitor the location of UreB and EEA1. 4′,6-diamidino-2-phenylindole (DAPI) was used to visualize the nuclei (0.1 mg/mL, Molecular Probes, Eugene, OR, United States).

For live cell experiments, AGS cells were previously transfected with Lamp1-GFP (donated by Dr. Allan Levey, Emory University) using Lipofectamine 2000 transfection reagent (Invitrogen) according to manufacturer’s recommendations. HPU was covalently tagged with Texas Red (Sulforhodamine 101 acid chloride; Sigma–Aldrich).

*Helicobacter pylori* urease incubated with Texas Red (0.5 mg/mL) during 1 h, at 4°C, with continuous stirring. This sample was then exhaustively dialyzed against 20 mM phosphate buffer, pH 7.0, and gel-filtrated using a De-Salting column (Sigma–Aldrich) to remove any excess of free dye.

### Cholesterol Depletion

Cholesterol was depleted in AGS cells by incubation with 5 mg/mL mβCD (Sigma–Aldrich) in serum-free medium at 37°C under 5% CO_2_ for 60 min (Hutton et al., 2010). This treatment did not affect cell viability as assessed by the trypan blue exclusion test (Strober, 2001).

### Cell Viability Assay

The viability of the gastric epithelial cells upon HPU treatment was evaluated using the MTT assay 3-(4,5-Dimethylthiazol-2-yl)-2,5-diphenyltetrazolium bromide (M2128) (Sigma–Aldrich), according to manufacturer’s instructions. Cells were seeded at a density of 1.5 × 10^5^ cells/well in 96-well plates, and then incubated with 300 nM HPU or PBS for 24 h. After incubation, 20 μL of MTT solution (5 mg/mL) was added to each well and incubated for 150 min at 37°C. The media was carefully removed and 150 μL MTT solvent (4 mM HCl, 0.1% NP40, in isopropanol) was added to the wells. The absorbance was measured at 570 nm in a plate reader (SpectraMax M3, Molecular Devices, Sunnyvale, CA, United States). The experiments were performed in triplicates.

### Analysis for Apoptosis-Related Proteins by Western Blot

Two different administrations schedules were used for HPU incubation in AGS cells. To investigate an “acute” effect of the protein, two doses of 300 nM HPU were added to the medium at 0 and 2 h, and the experiment was concluded after 6 h of total incubation. To simulate a “chronic” effect, cells received three doses of HPU, with additions of freshly diluted HPU (300 nM) at 0, 6 and 24 h time-points and the experiment was terminated after 48 h. Cell lysates from both groups were prepared, denatured in sample buffer (50 mM Tris-HCl, pH 6.8, 1% SDS, 5% 2-mercaptoethanol, 10% glycerol, 0.001% bromophenol blue) and heated in a boiling water bath for 3 min. Samples (30 μg total protein) were resolved in 10% SDS-PAGE gels and proteins were transferred to polyvinylidene difluoride (PVDF) membranes (Hybond-P, Amersham Pharmacia Biotech). Rainbow markers (Thermo Fisher Scientific, Inc.) were run in parallel to estimate molecular masses. Membranes were blocked with Tween-Tris buffered saline (TBS) (20 mM Tris-HCl, pH 7.5, 500 mM NaCl, 0.1% Tween-20) containing 1% bovine serum albumin (BSA) and probed with rabbit monoclonal antibodies: anti-Bcl-X_L_ (Cell Signaling Technology, Danvers, MA, United States, 1:500) and anti-BAD (Cell Signaling Technology, 1:500). Secondary antibodies (anti-rabbit, 1:20,000) coupled to horseradish peroxidase were from Jackson ImmunoResearch Laboratories, Inc. (West Grove, PA, United States). The protein bands were visualized using a chemiluminescence detection kit (Millipore, Billerica, MA, United States). The levels of protein expression were quantified using the software ImageJ and normalized against β-actin as an endogenous control.

### Human Angiogenesis-Related Proteins

The Human Angiogenesis Array Kit (Proteome Profiler^TM^- ARY007 Array, R&D Systems, Inc., Minneapolis, MN, United States) was used to detect the expression of angiogenesis-related proteins in cellular extracts from AGS cells treated with 300 nM HPU for 9 h in comparison with PBS-treated cells, as control. The array was performed once according to manufacturer’s instructions, using a pool of cellular lysates from three independent experiments, containing 300 μg of total protein.

### Endothelial Cell Capillary-Like Tube Formation Assay

Human umbilical vascular endothelial cells were seeded in 96-wells plates coated with 100 μL of growth factor-reduced Matrigel^TM^ (Corning^®^Inc., Tewksbury, MA, United States) in serum-free RPMI 1640 GlutaMax medium (Gibco) at a density of 6 × 10^4^ cells per well in the presence of 300 nM HPU or PBS (control) for a total of 9 h. HUVECs were allowed to stabilize for 3 h in a cell culture incubator at 37°C with 5% CO_2_ humidified atmosphere. The formation of endothelial network was then followed in the center of each well using a Leica DMI 6000 time-lapse microscope (Leica Microsystems, Germany) for 6 h, with a 10x magnification and z-stacks of 2.08 μm were acquired every 30 min. The number of tubes and branching points per microscopic field were automatically quantified using Angiogenesis analyzer plugin (Carpentier, 2012) for ImageJ software (Schneider et al., 2012).

### Chicken Embryo Chorioallantoic Membrane Angiogenesis Assay

The chicken embryo CAM model was used to evaluate angiogenic activity of HPU as previously described (Teresa Pinto et al., 2016). HPU (50 nM, *N* = 16; 100 nM, *N* = 12 and 500 nM, *N* = 14), 2.78 μM of b-FGF2 (positive control, *N* = 14) and PBS/vehicle (negative control, *N* = 16) were tested. Briefly, fertilized chick (*Gallus gallus*) eggs obtained from commercial sources were incubated horizontally at 37.8°C in a humidified atmosphere and referred as embryonic day (E). On E3, a square window was opened in the shell after removal of 2–2.5 mL of albumen to allow detachment of the developing CAM. The window was sealed with a transparent adhesive tape and the eggs returned to the incubator. On E10, 10 μL of test solution were placed on the top of growing CAM into a 3 mm silicon ring under sterile conditions. The eggs were re-sealed and returned to the incubator for three more days. After removing the ring, the CAM was excised from the embryos, photographed *ex ovo* under a stereoscope, at 20x magnification (Olympus, SZX16 coupled with a DP71 camera). The number of new vessels (less than 15 μm diameter) growing radially toward the ring area was counted in a blind fashion manner. Statistical analysis was performed as described below.

### Statistical Analysis

The statistical significance of the differences between two groups was assessed using the unpaired Student’s *t*-test and for multiple comparisons it was performed a two-way analysis of variance (ANOVA) followed by Dunnett’s multi-comparison post hoc test, used to calculate significance. GraphPad Prism6 software (San Diego, CA, United States) was used to perform statistical analysis. Statistically significance was set at p-value ≤ 0.05. Data in graphs represent average ± standard error of the mean (SEM) of at least three experiments, unless otherwise stated.

## Results

### HPU Is Not Cytotoxic to Gastric Epithelial Cells

**FIGURE 1:**
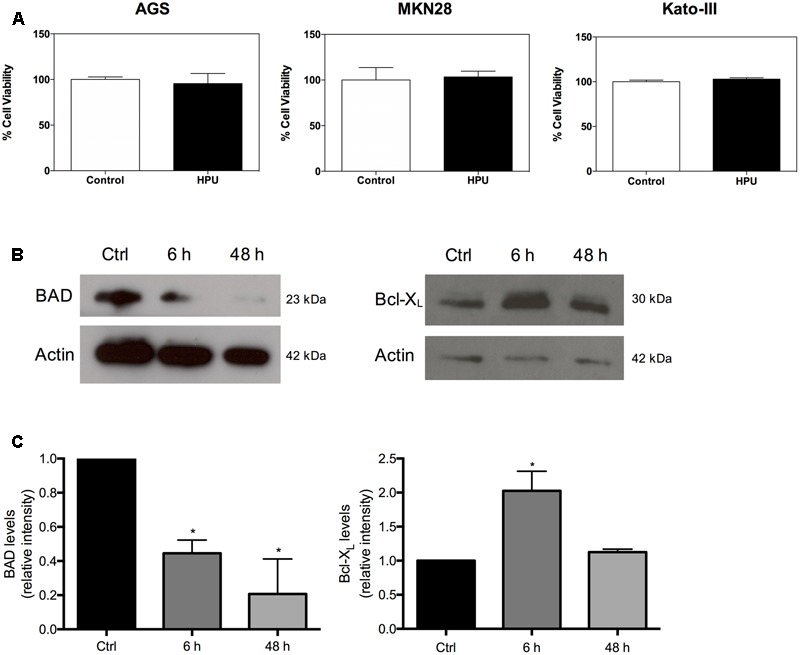
*Helicobacter pylori* urease is not cytotoxic to gastric epithelial cell lines. **(A)** Effect of HPU in cell proliferation and viability of AGS, Kato-III, and MKN28 cell lines incubated with 300 nM HPU or PBS for 24 h, was evaluated by the MTT assay. **(B)** Levels of pro- and anti-apoptotic proteins in AGS cells treated with daily freshly added 300 nM HPU for 6 h (“acute” exposure), and for 48 h (“chronic” exposure). **(C)** Cell lysates were analyzed for the cytoplasmic content of BAD (left panel) and Bcl-X_L_ (right panel) by Western blot. Results shown are means ± SEM, *N* = 3; ^∗^*p* ≤ 0.05 was considered statistically significant.

To address whether purified HPU could affect cell proliferation and viability of gastric epithelial cell lines we performed the MTT cell proliferation assay. The incubation of AGS, Kato-III, and MKN28 cells with 300 nM HPU for 24 h does not interfere with the proliferation rate, nor with the cell viability of gastric cell lines, as indirectly measured in the MTT assay (**Figure 1A**). Further, the levels of Bcl-X_L_, an anti-apoptotic protein, and the levels of BAD, a pro-apoptotic protein, were evaluated by western blot in AGS cells after incubation with HPU under two experimental conditions. One of them aimed to simulate an “acute” effect of HPU (6 h of treatment), and the other a “chronic” effect (48 h of treatment) (**Figures 1B,C**). HPU induced a significant decrease in the expression levels of BAD in both “acute” and “chronic” conditions when compared to untreated cells, of 55 and 80%, respectively. The levels of Bcl-X_L_, on the other hand, increased almost 100% relative to untreated cells, but only in the “acute” model of stimulation.

### HPU Is Internalized by Gastric Epithelial

**FIGURE 2:**
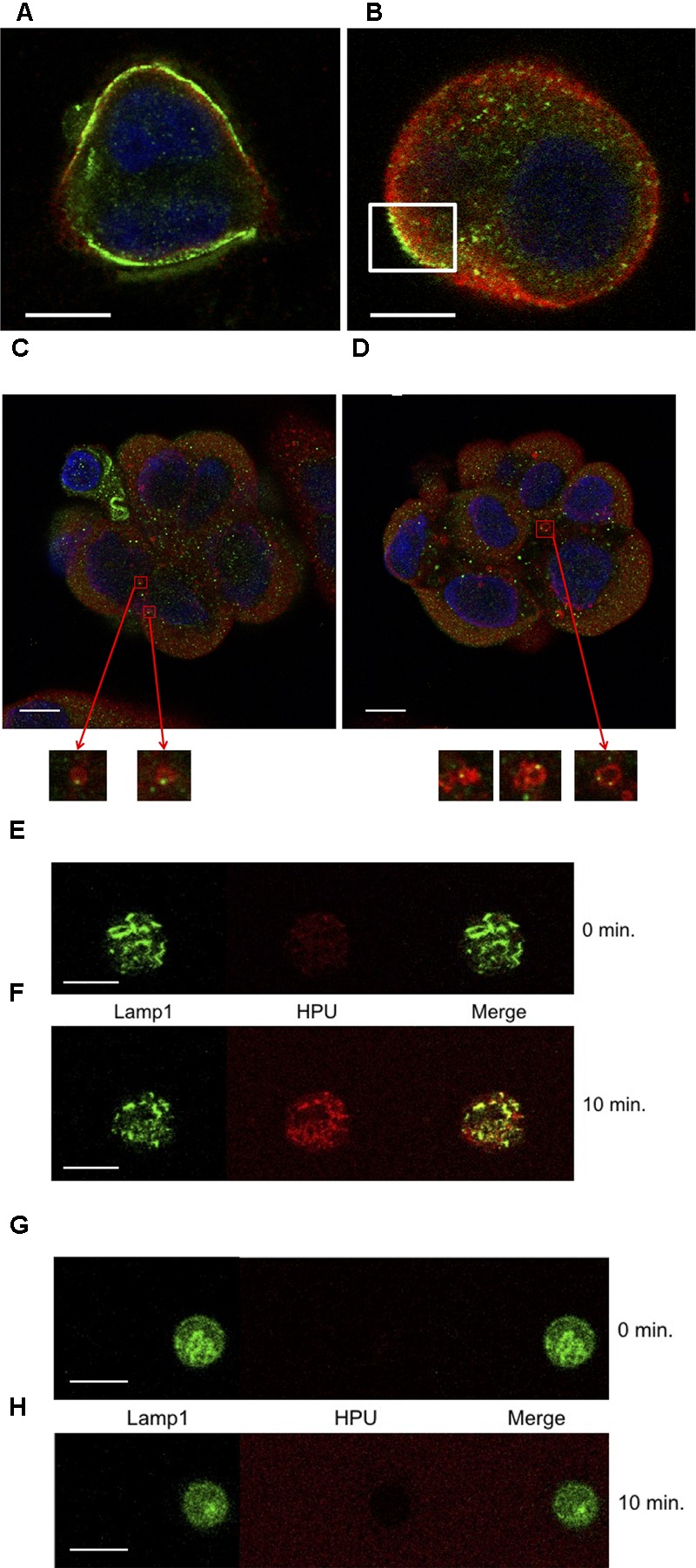
Internalization of HPU by gastric epithelial cells occurs through a classic endocytic pathway and is dependent on cholesterol. **(A,B)** Confocal immunofluorescence microscopy showing internalization of HPU in AGS cells treated with 100 nM HPU. Cells were fixed, permeabilized and processed for the detection of HPU, using an antibody against urease (Ure-B subunit) stained with Alexa-488-conjugated secondary antibody (green), and for the detection of early endosomes, using an antibody for EEA-1 with Alexa-596-conjugated antibody (red). A representative result is shown for cells fixed after 15 min **(A)** and after 45 min **(B)** of treatment with HPU, at 37°C. **(C,D)** Colocalization (yellow) of HPU (green) and EEA1-positive endosomes (red) in AGS cells treated with HPU for 5 min at 37°C evaluated by confocal microscopy. **(C,D)** Show different sections of the same cell agglomerate. Inserts are 100x original magnifications depicting EEA1-positive endosomes (anti-EEA-1 antibody and Alexa-596 secondary antibody) containing HPU (anti-UreB antibody labeled with Alexa-488 secondary antibody) next to the plasma membrane. **(E–G)** Endocytosis of HPU involves Lamp1-positive endosomes and is impaired upon cholesterol depletion. **(E,F)** AGS cells were transfected with a Lamp1-GFP plasmid prior to the addition of TexasRed-labeled HPU at 100 nM. **(G,H)** Transfected cells were pre-treated with 5 mg/mL mβCD, for 1 h, washed, and then treated with 100 nM TexasRed-labeled HPU. After approximately 1 min of contact of the cells with HPU, the detection of fluorescence by confocal microscope was started and the time point called 0 min. Scale bars 10 μm.

To explore if HPU is internalized by gastric epithelial cells, we treated AGS cells with HPU and performed confocal immunofluorescence microscopy. After 15 min of incubation, HPU detected with an anti-UreB antibody and an Alexa-488-conjugated secondary antibody was found on the plasma membrane, and some molecules were already in the cytoplasm (**Figure 2A**). After 45 min post-stimulation, HPU was still seen in a specific area of the plasma membrane (**Figure 2B**), suggesting that the internalization process may occur via specific receptors or cellular structures. Next, to gain insight of HPU’s internalization route, AGS cells were analyzed at shorter periods of treatment for co-localization of HPU and early endosomes, by confocal immunofluorescence microscopy. As shown in **Figures 2C,D**, during the initial steps of internalization HPU co-localizes with EEA1-positive early endosomes. After 10 min, a co-localization with Lamp1 was observed, suggesting the progression of HPU sorting to late endosomes/lysosomes (**Figures 2E,F**).

In order to investigate the involvement of cholesterol in the binding and endocytosis of HPU, AGS cells were treated with mβCD, a cholesterol scavenger, previously to treatment with HPU. **Figures 2G,H** show that binding of HPU to the cell membrane depends on cholesterol, suggesting that HPU internalization probably occurs through cholesterol-rich plasma membrane structures, such as lipid rafts.

### HPU Induces Angiogenesis

**FIGURE 3:**
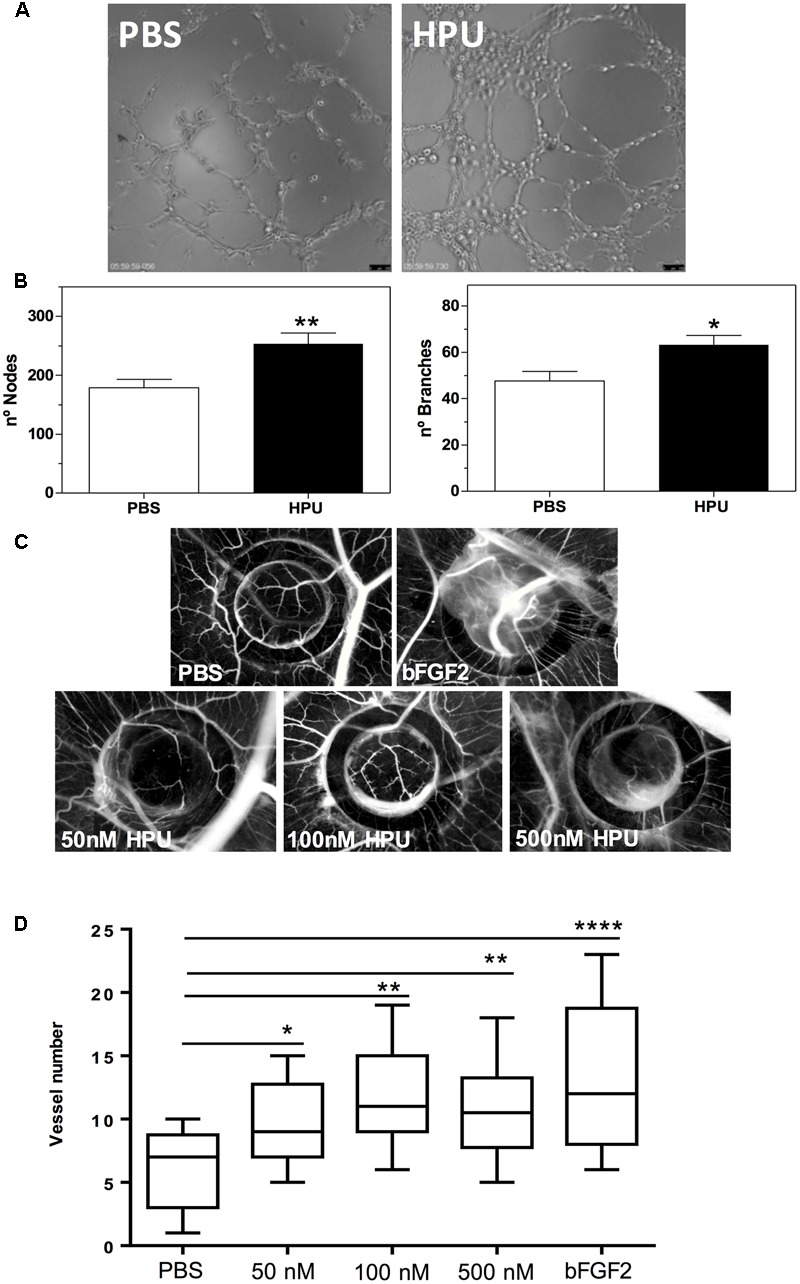
*Helicobacter pylori* urease induces an angiogenic response both *in vitro* and *in vivo* models. **(A,B)** Effect of HPU in the induction of tube-like structures by HUVECs. **(A)** Representative micrographs showing the endothelial network formed by HUVECs exposed to 300 nM HPU after 9 h of treatment in comparison with that of untreated cells. **(B)** Quantification of the number of nodes and branches points formed per microscopic field of three independent experiments, using the plugin Angiogenesis Analyzer from the ImageJ software. Data are presented as mean ± SEM, ^∗∗^*p* <0.005 and ^∗^*p* < 0.01 *versus* control, unpaired two-tail *t*-test. **(C,D)** Angiogenenic response induced by HPU on the *in vivo* CAM Assay. **(C)** Representative photomicrographs of new blood vessel formation are shown per condition (original magnification 20x). Analysis of HPU-induced angiogenesis was performed through quantification of the number of new vessels in controls and experimental conditions. **(D)** Number of new vessels in the CAM after 72 h incubation with 10 μL of 50, 100, and 500 nM HPU; PBS (negative control) and 10 μL of a 2.78 μM solution of b-FGF2 (positive control). Data regarding 12–16 fertilized eggs per condition is depicted on the box plot graph. ANOVA analysis, followed by Dunnett’s multi-comparison test, demonstrated significant differences between groups. ^∗∗∗∗^*p* < 0.0001, ^∗∗^*p* < 0.005, and ^∗^*p* < 0.05.

To investigate the involvement of HPU in angiogenesis we performed the endothelial cell tube formation assay. The addition of 300 nM HPU induced the formation of tube-like structure by HUVECs, after 9 h of treatment (**Figure 3A**). Quantification of the total number of tubes and branching points per microscopic field showed a significantly higher number of both structures in HPU-treated HUVECs than in control, the PBS-treated HUVECs (**Figure 3B**).

Further, to evaluate the role of HPU in an *in vivo* angiogenesis model we used the chick embryo CAM assay. All tested concentrations of HPU (50, 100, and 300 nM) induced a strong angiogenic response comparable to the positive control (b-FGF2) and a significantly higher angiogenic response than the negative control, as measured by the number of new blood vessels formed (**Figures 3C,D**). Together, the data from the *in vitro* and *in vivo* angiogenesis assays indicate that HPU can act as a pro-angiogenic factor in *H. pylori* infections.

### HPU Reshapes the Expression of Angiogenic Factors

**FIGURE 4:**
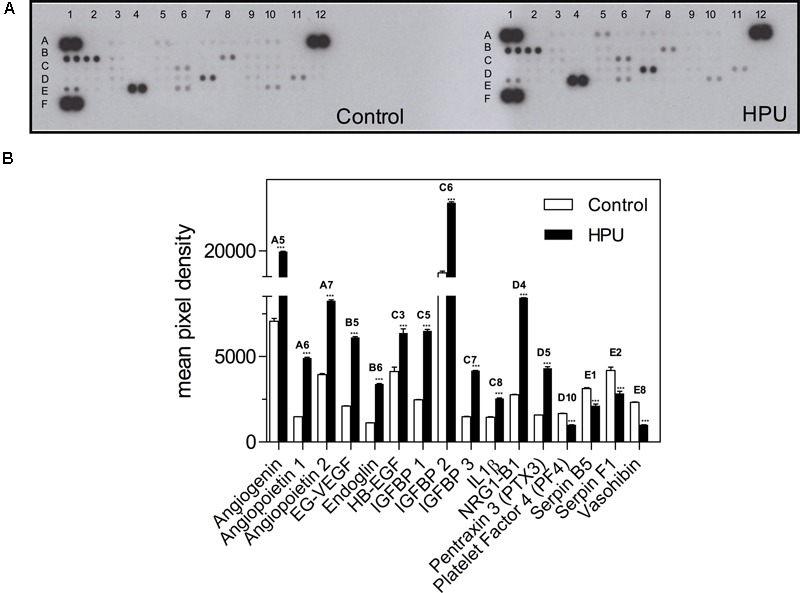
Effect of HPU on the expression levels of angiogenesis-related proteins. **(A)** Angiogenic array membranes obtained with cellular extracts from AGS cells treated or untreated with 300 nM HPU for 9 h. **(B)** Profile of angiogenic factors generated from the array membranes. The values in the bar graph represent the mean of pixel density of the two spots of the array ±SEM from one experiment, using pooled cellular extracts from three independent experiments; ^∗∗∗^*p* < 0.001 *versus* control, unpaired two-tailed *t*-test.

To characterize the angiogenic profile of gastric epithelial cells upon treatment with HPU we performed an antibody angiogenesis array, using cellular extracts of AGS cells treated with 300 nM HPU and its untreated counterparts, pooled from three independent experiments. The effect of treatment of AGS cells with HPU on the expression of molecules involved in angiogenesis was then analyzed. The increased expression of angiogenin, angiopoietin-1, angiopoietin-2, EG-VEGF, endoglin, HB-EGF, IGFBP-1, IGFBP-2, IGFBP-3, IL-1β, neuroglin-B1 (NRG1-B1) and pentraxin-3 (PTX3), well-known pro-angiogenic factors and the decrease of anti-angiogenic factors platelet factor 4 (PF4), serpin B5, serpin F1 and vasohibin, were detected in the array assay, confirming the participation of HPU in angiogenesis-related processes (**Figure 4**).

## Discussion

Estimates are that *H. pylori* infection accounts for 5.5% gastroduodenal cancer worldwide and for more than 60% of all gastric cancer cases (Correa and Piazuelo, 2011). The infection by *H. pylori* causes damage of gastric tissues in humans, with epithelial cell cytoplasmic vacuolization and disorganization of gastric glands in the mucosa (Kusters et al., 2006). Ulcers due to gastritis in *H. pylori* infected patients have a typically slow healing process (Brzozowski et al., 1999). Previous studies using different models of endothelial cells *in vitro* have indicated that angiogenesis, a process that enhances microcirculation and is critical to recover to wounded tissue, is impaired by *H. pylori* infection (Kim et al., 2004; Tobin et al., 2008). Kim et al. (2004) have demonstrated that treatment of HUVECs with a water extract of *H. pylori* significantly inhibited capillary tube formation and decreased the expression of VEGF and angiopoietin.

Several damages seen in gastric tissues infected with *H. pylori* have been ascribed to the vacuolating toxin, VacA. For example, Tobin et al. (2008) reported that a conditioned medium obtained from a VacA-positive *H. pylori* strain inhibited bovine aortic endothelial cell functions through a VacA-dependent nitric oxide reduction mechanism. This conditioned medium reduced cell proliferation, tube formation and migration, effects that were blocked by the VacA inhibitor 5-nitro-2-(3-phenylpropylamino)benzoic acid. Moreover, no such effects were observed in cells treated with a VacA-negative *H. pylori-* or *Escherichia coli*-conditioned media (Tobin et al., 2008). VacA is known to associate with lipid rafts and is internalized by gastric cells (Gauthier et al., 2005). Moreover, VacA is known to reach the cytoplasm and promote vacuolization and affect mitochondrial function in gastric epithelial cells by mechanisms not yet fully characterized (Palframan et al., 2012).

On the other hand, other *H. pylori*-derived proteins promote angiogenesis and contribute to tumor invasiveness and growth. *H. pylori* heat shock protein 60 (HSP60) has been reported to play a role in tumor aggressiveness (Li et al., 2014) and to display proangiogenic activity in HUVECs (Lin et al., 2010). Cyclooxygenase-2 has also been implicated in angiogenesis and tumor invasiveness due to *H. pylori* (Chang et al., 2005). Here we demonstrate that HPU, a crucial virulence factor that allows bacterial survival and colonization of gastric mucosa, also plays a role in processes that underlie tumor growth in the stomach of infected patients.

Searching for a better understanding of how HPU exerts its pro-angiogenic effects, we have monitored the cytotoxicity of the purified urease on three gastric cell lines and studied its internalization by AGS cells. Incubation with 300 nM HPU for 24 h has not interfered in cell proliferation rates or in the cell viability of AGS, Kato-III, and MKN28 cells. This is a relatively high protein concentration considering the dose range (10–500 nM) of HPU which activates other cell types, such as platelets (Wassermann et al., 2010) and neutrophils (Uberti et al., 2013).

HPU’s internalization is evident about 5 min following its addition to AGS cells. This internalization seems to be a receptor- or structure-specific process since we could observe an increased density of HPU-immunoreactive molecules in a particular region of the plasma membrane after 45 min of incubation, when most of the protein had already been endocytosed (**Figure 2B**). The lack of binding and internalization of HPU after treatment of AGS cells with the cholesterol scavenger mβCD indicates that specific plasma membrane regions, such as cholesterol-rich lipid rafts could be involved in this process. It has been reported that *H. pylori* takes advantage of cholesterol-rich lipid rafts to induce responses in epithelial cells, such as the activation of NF-κB and expression of IL-8 (Hutton et al., 2010). Particularly relevant to *H. pylori* pathogenesis is its type IV secretion system, which delivers CagA and VacA into the gastric cell and interacts with lipid rafts (Schraw et al., 2002; Lai et al., 2008).

As shown in **Figures 2C,D**, HPU co-localized with early endosomes, positives for the marker EEA1. Early endosomes are the first endocytic compartment to accept incoming cargo after internalization from the plasma membrane and their primary function is the sorting of internalized molecules to different intracellular locations (Marko et al., 2010).

Lamp-1 is a marker found in either late endosomes or lysosomes, as a glycoprotein present in the cytoplasmic face of these organelles (Cook et al., 2004). **Figure 2F** shows that 10 min after the addition of HPU to AGS cells, the protein could be localized in structures containing Lamp-1, suggesting that the urease may be directed to a degradation route. The intracellular fate of HPU beyond this point was not investigated in this study. On the other hand, as an invasive bacterium, *H. pylori* induces autophagy in gastric epithelial cells, a process that results in the sequestration of cytosolic components within double-membrane vesicles called autophagosomes (Terebiznik et al., 2009). These vesicles fuse with lysosomes to become autophagolysosomes, in which the vesicle contents are degraded by lysosomal hydrolases. *H. pylori* was reported to modulate and subvert this process preventing the formation of autophagolysosomes, thereby allowing bacterial proliferation (Greenfield and Jones, 2013). It has also been described that *H. pylori* induces the fusion of phagosomes, generating large less-acidic compartments called megasomes, which are necessary for intracellular bacterial survival. The formation of megasomes was shown to be HPU-dependent (Schwartz and Allen, 2006). As suggested by the HPU-like immunoreactivity localized in Lamp1-positive compartments, the protein itself or fragments of it after lysosomal hydrolysis, may have a role in the regulation of the fusion of lysosomes and autophagosomes.

The strong inflammatory response of gastric epithelial cells to *H. pylori* infection results in an intense infiltration of the gastric mucosa by polymorphonuclear leukocytes, macrophages, and lymphocytes. The degree of mucosal damage correlates with the degree of neutrophil infiltration (D’Elios et al., 2007). *H. pylori* was shown to induce apoptosis of gastric epithelial cells both *in vivo* and *in vitro* (Cover et al., 2003). Our group has previously shown that HPU activates human neutrophils and delays their apoptosis by altering the expression of pro- and anti-apoptotic proteins (Uberti et al., 2013). Here, we showed that purified HPU tested on three different gastric cancer epithelial cell lines increased their survival and inhibited apoptosis by modulation of the levels of Bcl-X_L_, an anti-apoptotic protein, and of BAD, a pro-apoptotic protein. This result goes in line with the hypothesis of a cancer promoting effect of HPU, that could add to or potentiate the action of other tumor-inducing factors produced by the bacterium.

The production of high cytokine levels by gastric cells upon *H. pylori* infection has been reported (Fiorentino et al., 2013), accompanied by activation of matrix metalloproteinase 10 via EGFR and ERK-mediated pathways (Costa et al., 2016). To evaluate the participation of HPU in tumorigenic processes related to *H. pylori*, we investigated the role of this protein in angiogenesis by measuring the expression of molecules involved in this phenomenon using an array analysis, and *in vitro* tubulogenesis and *in vivo* angiogenesis models.

The capillary-like tube forming assay and the Chicken embryo CAM assay are two well-accepted *in vitro* and *in vivo* models in angiogenesis studies (Kim et al., 2004; Teresa Pinto et al., 2016). Platelet factor-4 (PF-4/CXCL4) was reported to inhibit angiogenesis using the CAM assay (Maione et al., 1990). Studies demonstrating down-regulation of VEGF and angiopoietin receptors in HUVECs by *H. pylori* employed a capillary tube formation assay (Kim et al., 2004). Here, we showed that purified HPU induced angiogenesis in both *in vitro* and *in vivo* models. Addition to the CAM of 1–5 picomoles of HPU produced a strong angiogenic effect equivalent to that obtained for 27.8 picomoles of b-FGF2, the positive control (**Figure 3D**). Thus, the angiogenic stimulus provided by HPU is about 10–15-fold more potent than that of b-FGF2.

It has been shown that the array analysis of molecules participating in angiogenesis in AGS cells after treatment with 300 nM HPU led to an increased expression of pro-angiogenic factors such as angiogenin (Miyake et al., 2015), angiopoietins 1 and 2 (Jones et al., 2001; Zhou et al., 2009), EG-VEGF, also known as prokineticin-1 (Goi et al., 2004; Alfaidy et al., 2014), HB-EGF (Ito et al., 2001; Mehta et al., 2008), endoglin (Ollauri-Ibanez et al., 2017), IGFBP 1, 2 and 3, considered as endothelial tumor markers (Hintz et al., 1991; Lee et al., 2000; Le Jan et al., 2006), IL-1β (Kaluz and Van Meir, 2011; Christofides et al., 2015), NRG1-B1 (Gemberling et al., 2015), and PTX3 (Stallone et al., 2014). The same experiment showed contrasting results regarding the expression of anti-angiogenic factors in HPU-treated AGS cells, revealing decrease in the levels of PF-4 (Maione et al., 1990; Vandercappellen et al., 2011), serpin B5 and serpin F1 (Cher et al., 2003; Law et al., 2006), and vasohibin-1 (Sato, 2013).

## Conclusion

The set of results presented here allow us to conclude that *H. pylori*’s urease, a well-recognized virulence factor for its enzymatic property that allows bacterial survival in the stomach mucosa, displays a potent and so far overlooked pro-angiogenic activity on gastric epithelial cells. Altogether with its platelet- and neutrophil-activating properties and pro-inflammatory activity, which are independent of its enzyme nature, HPU has potential to contribute in other ways to the pathogenesis of diseases caused by *H. pylori*. The data also emphasize the multifunctional character of this protein and throw light on the need of new approaches to better understand the pathologies associated with *H. pylori*.

## Author Contributions

DO-S: planning and conducting the angiogenesis experiments; AU: planning and conducting the cell viability and internalization experiments; MSM: conducting immunofluorescence and cell transfection with GFP-Lamp1 plasmid; MTP: conducting and analysis of CAM assay; MG-L: conducting immunofluorescence experiments; DO-S, AU, CF, ML, and CC: have written and revised the manuscript; ML (in Portugal) and CC (in Brazil) have coordinated this study.

## Conflict of Interest Statement

The authors declare that the research was conducted in the absence of any commercial or financial relationships that could be construed as a potential conflict of interest.

## Abbreviations

b-FGF2: fibroblast growth factor
CAM: chorioallantoic membrane model
EG-VEGF: endocrine gland-derived vascular endothelial growth factor
GFP: green fluorescent protein
HB-EGF: heparin- binding epidermal growth factor-like growth factor
HPU: *Helicobacter pylori* urease
HUVECs: human umbilical vascular endothelial cells
IGFBP: insulin-like growth factor-binding proteins
IL-1β: interleukin 1β
mβCD: methyl-β-cyclodextrin
MTT: 3-(4,5-dimethylthiazol-2-yl)-2,5-diphenyltetrazolium bromide
NRG1-B1: neuroglin
PF-4: platelet factor-4
PTX3: pentraxin-3
TSAII: trypticase soy agar with 5% sheep blood
VEGF: vascular endothelial growth factor
